# Gene expression analysis of *Alcaligenes faecalis* during induction of heterotrophic nitrification

**DOI:** 10.1038/s41598-021-02579-3

**Published:** 2021-11-29

**Authors:** Shuhei Tsujino, Hideo Dohra, Taketomo Fujiwara

**Affiliations:** 1 Department of Environment and Energy Systems, Graduate School of Science and Technology, Shizuoka University, 836 Oh-ya, Suruga-ku, Shizuoka, 422-8529 Japan; 2 Instrumental Research Support Office, Research Institute of Green Science and Technology, Shizuoka University, Shizuoka, Japan; 3 Department of Science, Graduate School of Integrated Science and Technology, Shizuoka University, Shizuoka, Japan

**Keywords:** Biochemistry, Genetics, Microbiology

## Abstract

*Alcaligenes faecalis* is a heterotrophic nitrifying bacterium that oxidizes ammonia and generates nitrite and nitrate. When *A. faecalis* was cultivated in a medium containing pyruvate and ammonia as the sole carbon and nitrogen sources, respectively, high concentrations of nitrite accumulated in the medium whose carbon/nitrogen (C/N) ratio was lower than 10 during the exponential growth phase, while the accumulation was not observed in the medium whose C/N ratio was higher than 15. Comparative transcriptome analysis was performed using nitrifying and non-nitrifying cells of *A. faecalis* cultivated in media whose C/N ratios were 5 and 20, respectively, to evaluate the fluctuations of gene expression during induction of heterotrophic nitrification. Expression levels of genes involved in primary metabolism did not change significantly in the cells at the exponential growth phase under both conditions. We observed a significant increase in the expression levels of four gene clusters: *pod* cluster containing the gene encoding pyruvic oxime dioxygenase (POD), *podh* cluster containing the gene encoding a POD homolog (PODh), *suf* cluster involved in an iron-sulfur cluster biogenesis, and *dnf* cluster involved in a novel hydroxylamine oxidation pathway in the nitrifying cells. Our results provide valuable insight into the biochemical mechanism of heterotrophic nitrification.

## Introduction

Some heterotrophic bacteria possess the biological ability to oxidize ammonia and to form nitrite or nitrate. Generally, the nitrifying activity per cell of heterotrophic bacteria is very low compared to that of the autotrophic nitrifying microorganisms. However, due to their large biomass and species richness in soil, overall nitrifying activity by heterotrophic bacteria cannot be ignored, especially in acidic soils such as coniferous forests where the nitrification by autotrophic microorganisms is inhibited^1–3^. The biological activity of heterotrophic nitrification has been reported mainly in soil bacteria of the phylum Proteobacteria, but some species of actinomycetes and ascomycetes have been also known to exhibit nitrifying activity^4,5^. In addition, nitrogen removal by a combination of heterotrophic nitrification and aerobic denitrification is a promising technology for efficient wastewater treatment and has been actively studied^6–8^. In contrast, the biochemical mechanism of heterotrophic nitrification and its regulatory system have remained poorly understood.

Two different biochemical pathways are known in nitrification by heterotrophic microorganisms. The first is a reaction pathway similar to that of autotrophic ammonia-oxidizing bacteria and archaea (AOB and AOA), in which ammonia is oxidized to nitrite via hydroxylamine by the successive catalytic actions of ammonia monooxygenase (AMO) and hydroxylamine oxidoreductase (HAO). This type of HAO, which has been reported from some bacteria in the genera *Paracoccus*, *Acinetobacter*, and *Pseudomonas*, is not a high-molecular-weight multiheme protein present in the autotrophic AOB but is an enzyme containing a nonheme iron as the prosthetic cofactor^9–13^. However, the genetic, catalytic, and molecular properties of this nonheme iron-type HAO remain obscure.

The second nitrification proceeds via a unique pathway in which oxime or nitro compounds are the intermediate metabolites^14–16^. *Alcaligenes faecalis* is one of the bacteria that perform this type of nitrification and whose heterotrophic nitrification has been subjected to study^17,18^. Ono et al.^19^ purified the pyruvic oxime dioxygenase (POD), which catalyzes the dioxygenation of pyruvic oxime or oxaloacetic oxime to produce nitrite, from *A. faecalis*. Ono et al*.*^20^ further proposed a pathway of heterotrophic nitrification where the hydroxylamine generated by the AMO reacts with pyruvate non-enzymatically; then the pyruvic oxime thus generated is decomposed by POD into nitrite and pyruvate. Subsequently, it has been shown that the *pod* gene encoding the enzyme is widely distributed in the bacteria of the phyla Proteobacteria and Actinobacteria, as well as in the eukaryotic microorganism of the phylum Ascomycota^21^.

It has been reported that nitrifying activity is strongly induced when using a synthetic medium containing organic acids, especially pyruvate and acetate, as carbon sources^19,22,23^. In this study, cultivation experiments of *A. faecalis* were conducted using a synthetic medium containing organic acid and ammonium as the sole carbon and nitrogen sources, respectively, to elucidate the medium composition appropriate for the induction of nitrifying activity. It was confirmed that nitrification was induced in the bacterial cells when cultivated in a pyruvate-containing medium with a low carbon/nitrogen (C/N) ratio, as was suggested previously^23^. Comparative transcriptome analysis using the *A. faecalis* cells cultivated under the low and high C/N ratio conditions, where the nitrifying activity is induced or not induced, respectively, suggested the involvement of four gene clusters in the heterotrophic nitrification process. The biochemical mechanism of the heterotrophic nitrification in *A. faecalis* is discussed based on the genetic framework provided by this study.

## Results and discussion

**Figure 1:**
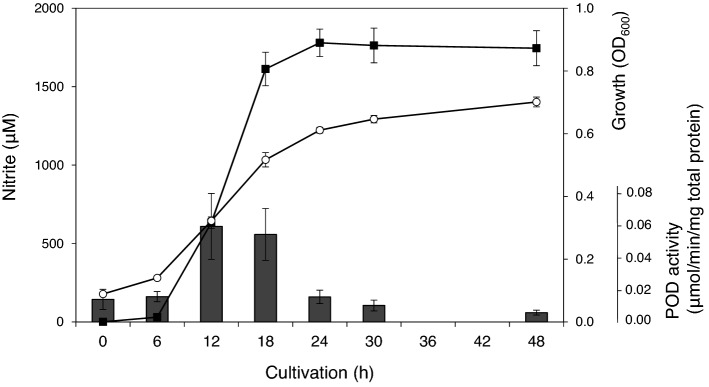
Cultivation of *A. faecalis* and induction of POD activity. *A. faecalis* was cultivated in a synthetic medium containing ammonium chloride and sodium pyruvate as the only nitrogen and carbon source. The culture medium was sampled over time, and the OD_600_ (open circles) was measured. The concentration of nitrite in the culture medium (closed squares) and the specific POD activity per protein (bars) were measured, respectively, after removing bacteria cells from the culture medium by centrifugation. Experiments were performed independently three times. Error bars represent standard error (S.E.).

*Alcaligenes faecalis* was cultivated in a synthetic medium containing 10 mM sodium pyruvate and 8 mM ammonium chloride as sole carbon and nitrogen sources, respectively, and the concentration of nitrite in the medium and the POD activity of the bacterial cells was measured. As shown in Fig. 1, the concentration of nitrite in the medium increased during the exponential growth of the bacterium. Twenty-four hours after starting cultivation, the concentration of nitrite accumulated in the medium reached 1.8 mM, which corresponds to 22% of the initial ammonium concentration. The concentration became almost constant in the subsequent stationary phase. On the other hand, POD activity in the bacterial cells reached the maximum in the late exponential growth phase, then rapidly decreased in the subsequent stationary phase. It has been known that the purified POD from *A. faecalis* is rapidly inactivated even under ice cooling because the cofactor divalent iron is easily shed from the enzyme molecule^21^. The rapid decrease of POD activity during the stationary phase may be due to the lack of intracellular iron.

**Figure 2:**
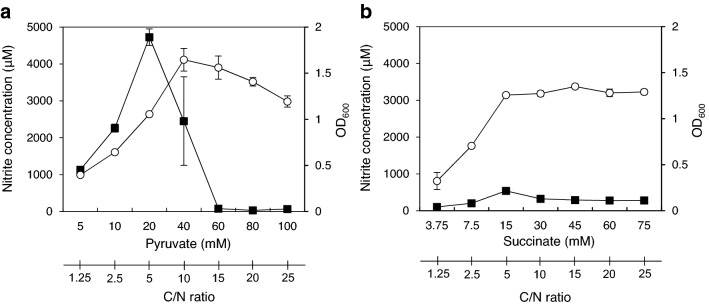
Effects of organic acids on nitrite accumulation in *A. faecalis*. *A. faecalis* was cultivated in a synthetic medium containing 10 mM ammonium chloride as the only nitrogen source and various concentrations of pyruvate (**a**) or succinate (**b**) as the only carbon source, respectively. After 24 h of incubation (the stationary phase), OD600 (open circles) and nitrite concentration (closed squares) of the medium were measured. Experiments were performed independently three times. Error bars represent S.E.

*Alcaligenes faecalis* was cultivated in a synthetic medium containing 10 mM ammonium chloride as a nitrogen source and various concentrations of sodium pyruvate (5–100 mM) or sodium succinate (3.75–75 mM) as a carbon source to change the C/N ratio (1.25–25). Regardless of the composition of the media, the bacterial cells shifted from the exponential growth phase to the stationary one within 21 to 24 h after starting the cultivation (data not shown). Optical density values at 600 nm (OD_600_) of the respective medium after 24 h cultivation and the nitrite concentration accumulated are shown in Fig. 2. When pyruvate was used as the carbon source, high concentrations of nitrite accumulated in the medium containing less than 40 mM pyruvate (C/N ratio < 10), and reached the maximum at 20 mM pyruvate (C/N ratio = 5), whereas only a little accumulation was observed in the medium containing more than 60 mM pyruvate (C/N ratio > 15) (Fig. 2a). On the other hand, in the synthetic medium containing succinate, nitrite accumulation increased with increasing succinate concentration and reached a maximum value (about 540 µM) at 15 mM, while accumulation of about 200–300 µM nitrite was also observed at the higher concentrations ranging from 30 to 75 mM (Fig. 2b). The result indicated that, unlike pyruvate, succinate did little to inhibit accumulation of nitrite even at the high concentration. Nishio et al*.*^23^ reported that pyruvate and oxaloacetate were effective for induction of nitrifying activity of *A. faecalis* strain OKK17, but acetate, succinate, and malate were not. The results of this experiment indicated that, in the *A. faecalis* strain NBRC13111 also, nitrification activity is strongly induced in the synthetic medium which contains pyruvate as a carbon source but that the addition of excessive pyruvate inhibits the induction of the nitrification.

**Figure 3:**
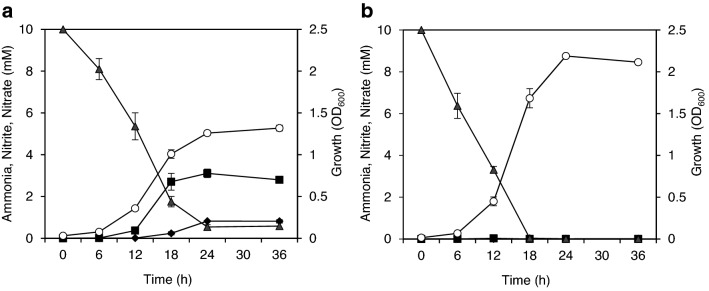
Effect of pyruvate on induction of heterotrophic nitrification in *A. faecalis*. *A. faecalis* was cultivated in a synthetic medium containing 10 mM ammonium chloride as the only nitrogen source or 20 mM (**a**) or 80 mM (**b**) sodium pyruvate as the only carbon source, respectively. The culture medium was sampled over time, and OD600 (open circles) was measured. The concentrations of ammonia (closed triangles), nitrite (closed squares), and nitrate (closed diamonds) in the medium were measured, respectively, after removing bacteria cells from the culture medium by centrifugation. Experiments were performed independently three times. Error bars represent S.E.

As shown in Fig. 3, temporal changes of ammonia, nitrite, and nitrate concentrations in the medium were followed during the cultivation of *A. faecalis* by using two synthetic media containing 10 mM ammonium chloride and low (20 mM) or high (80 mM) concentrations of sodium pyruvate. In the synthetic medium containing 20 mM pyruvate (C/N ratio = 5), 94% of the initial ammonia was consumed by 24 h after starting cultivation, and 33% and 9% of ammonium were converted to nitrite and nitrate, respectively, and then were accumulated in the medium (Fig. 3a). The doubling time (*t*_D_) was estimated to be 4.0 h. When using a medium containing 80 mM pyruvate (C/N ratio = 20), the initial ammonia was almost completely consumed after 24 h cultivation, and only trace amounts of nitrite and nitrate were detected in the medium (Fig. 3b). The *t*_D_ value was estimated to be 3.2 h in this cultivation. In both conditions, the bacterial cells in the exponential growth stage shifted to the stationary phase about 24 h after starting incubation.

The experimental results shown in Figs. 1, 2, 3 demonstrate that when *A. faecalis* is cultivated in the medium of low C/N ratio containing a low concentration of pyruvate, the accumulation of nitrite is caused by the POD activity induced in the bacterial cells in the exponential growth phase, and that the POD activity is greatly reduced in the bacterial cells in the stationary phase. It was also shown that accumulation of nitrite did not occur even in the exponential growth phase when using a medium of a high C/N ratio containing a high concentration of pyruvate. However, because the production of nitrous oxide (N_2_O) and dinitrogen (N_2_) gases was not measured in the experiments, it is unclear whether the reason why nitrite did not accumulate was that POD activity was not induced in the condition, or that the nitrite produced by nitrification was promptly consumed by aerobic denitrification. We conducted comparative transcriptome analysis to clarify the changes in the expression of *A. faecalis* genes affected by the cultivation condition.

*A. faecalis* was cultivated using two synthetic media, one containing 5 mM ammonium and 10 mM pyruvate, and the other containing 5 mM ammonium and 40 mM pyruvate. Bacterial growth and accumulation of nitrite in the former “Low C/N” medium whose C/N ratio was 5 (supplementary Fig. S1a), and in the latter “High C/N” medium whose C/N ratio was 20 (supplementary Fig. S1b), proceeded in almost the same manner as those indicated in Fig. 3a,b, respectively. The bacterial cells in the mid-exponential growth phase, designated “LowC/Nexp” cells, and those in the stationary phase, designated “LowC/Nsta” cells, were collected from the low C/N medium at 18 h and 28 h after starting cultivation, respectively. *A. faecalis* cells were also cultivated in the high C/N medium; then the bacterial cells at the mid-exponential growth phase were collected at 18 h after starting cultivation and designated “HighC/Nexp” cells. Total RNAs extracted from these three samples were used to prepare an RNA-seq library, and a total of approximately 56.5 million reads of transcriptome sequences were generated by paired-end sequencing. The RNA-seq data are summarized in supplementary Table S1. After mapping the filtered reads to the reference genome, the number of reads was counted and normalized to calculate the transcripts per million (TPM).

The difference between gene expressions of a total of 3,719 genes, except rRNA, of the LowC/Nexp cells and the HighC/Nexp cells was analyzed. Compared with the HighC/Nexp cells, 33 genes were up-regulated (log2 fold change (logFC) ≥ 2.0 and had a false discovery rate (FDR) < 0.05), and 72 genes were down-regulated (logFC ≤ -2.0 and had an FDR < 0.05), as listed in supplementary Table S2. Differences in gene expression are also shown by MA plotting in supplementary Fig. S2a. There were few genes involved in the primary metabolic pathways in the total 105 genes whose expression levels showed significant differences between LowC/Nexp cells and HighC/Nexp cells. For example, expression levels of genes (AFA2_00443-00445) encoding pyruvate dehydrogenase which links the glycolytic pathway with a tricarboxylic acid cycle, genes (AFA2_02578-02591) encoding NADH dehydrogenase, which is the start point of the respiratory electron transfer pathway, and genes (AFA2_02307-02314) encoding the FoF1-ATP synthase responsible for oxidative phosphorylation did not differ between the LowC/Nexp and the HighC/Nexp cells. These results suggested that, at least in the exponential growth phase, there is no significant change in the primary metabolic pathway for energy generation of *A. faecalis* cells cultivated under the Low C/N condition and the High C/N condition.

Of the 105 genes that showed differential expression between LowC/Nexp cells and HighC/Nexp cells, 19 and 9 genes were highly expressed (TPM > 1000) in the LowC/Nexp cells or the HighC/Nexp cells, respectively, as listed in Table 1. The *pod* gene (AFA2_01040) was strongly activated in the LowC/Nexp cells as expected. Besides the *pod* gene, there are three genes (AFA2_00520, AFA2_01076, AFA2_02258) encoding class II aldolase on the *A. faecalis* genome that are highly homologous to the *pod* gene. One of the three genes, AFA2_02258, which is designated *podh* (*pod homolog*) hereafter, was also greatly up-regulated in the LowC/Nexp cells as in the case of the *pod* gene. Furthermore, all the genes involved in both of two putative polycistronic transcription units, one of which consisted of five genes including the *pod* gene (AFA2_01038-01042), designated a *pod* gene cluster hereafter, and another consisted of four genes including the *podh* gene (AFA2_02256-02259), designated a *podh* gene cluster, were activated or showed a similar trend in the LowC/Nexp cells, as shown in Table 1 and supplementary Fig. S2. All seven genes (AFA2_00632-00638) in the *suf* gene cluster, which is involved in the iron-sulfur cluster biogenesis by the SUF (sulfur formation) system, were also up-regulated in the LowC/Nexp cells (Table 1). Significant increases in the expression of the three consecutive genes (AFA2_03346-03348) were detected in the LowC/Nexp cells. The AFA2_03346 gene encoding a novel hydroxylamine oxidase that oxidizes hydroxylamine to N_2_ was reported recently for the heterotrophic nitrifying bacterium *Alcaligenes* sp. HO-1^24^. The enzyme, designated DnfA, is the catalytic core of the DNF (dinitrogen formation) system encoded by *dnfABCD* gene cluster^24^. The three genes (AFA2_03346-03348), which are homologous to *dnfABC*, were activated in the LowC/Nexp cells with high TPM values, and the remaining *dnfD*-homologous gene (AFA2_03349) was also up-regulated and the TPM values reached 635 in the LowC/Nexp cells (Table 1 and supplementary Fig. S2). In contrast, four of nine up-regulated genes in the HighC/Nexp cells were presumed to be involved in the sulfur metabolism; phosphoadenosine phosphosulfate reductase (AFA2_03446) and sulfate adenylyltransferase (AFA2_03447) are the enzymes involved in the assimilatory sulfate reduction pathway, and the other two genes (AFA2_00269 and AFA2_02155) may participate in the cellular transport of sulfate or thiosulfate. Expression of a flagellin gene (AFA2_02843) was also elevated in the HighC/Nexp cells compared with that in the LowC/Nexp cells. Changes in the expression levels of the denitrifying genes encoding nitrite reductase NirK (AFA2_02352), nitric oxide reductase NorB (AFA2_02353), and N_2_O reductase NosZ (AFA2_02246) were not detected because their TPM values were maintained at high levels (higher than 1000) in both the LowC/Nexp and the HighC/Nexp cells.

**Table 1:** Major differentially expressed genes in LowC/Nexp cells compared to HighC/Nexp cells.

Locus tag	Product	logFC	FDR	TPM LowC/Nexp	TPM HighC/Nexp
AFA2_00269	Sulfate/thiosulfate transport system substrate-binding protein	− 3.13	0.00097	156.6	1374.8
AFA2_00279	Cyclohexyl-isocyanide hydratase	2.76	0.00497	2120.2	315.0
AFA2_00632	Fe-S assembly SUF system protein SufT	2.53	0.01075	1525.0	265.7
AFA2_00633	Fe-S cluster assembly scaffold protein SufU	2.77	0.00491	1299.3	192
AFA2_00634	Cysteine desulfurase/selenocysteine lyase SufS	2.63	0.00745	1322.2	214.0
AFA2_00635	Fe-S cluster assembly protein SufD	2.55	0.00982	1453.8	249.6
AFA2_00636	Fe-S cluster assembly ATP-binding protein SufC	2.57	0.00909	2278.8	384.7
AFA2_00637	Fe-S cluster assembly protein SufB	2.35	0.02274	1460.5	287.5
AFA2_00638	Transcriptional regulator SufR	2.36	0.02255	1811.0	354.2
AFA2_00700	Polar amino acid transport system substrate-binding protein	− 2.28	0.02937	282.6	1374.7
AFA2_00794	yggS family pyridoxal phosphate enzyme	3.30	0.00044	2028.2	207.4
AFA2_00795	Cytoplasmic protein	3.61	0.00012	2262.4	186.1
AFA2_01038	2-Hydroxychromene-2-carboxylate isomerase	3.43	0.00024	1732.9	161.0
AFA2_01039	MHS family, shikimate and dehydroshikimate transport protein	3.59	0.00013	1422.4	119.1
AFA2_01040	Pyruvic oxime dioxygenase POD	4.29	2.85E−06	4620.4	237.5
AFA2_01041	Hypothetical protein	4.69	5.42E−07	1611.9	62.8
AFA2_01137	Cytochrome *c* peroxidase	− 2.64	0.00738	309.5	1936.3
AFA2_02155	Thiosulfate transporter subunit	− 2.84	0.00344	277.4	1990.4
AFA2_02256	Putative tricarboxylic transport membrane protein	4.52	9.56E−07	1013.4	44.4
AFA2_02258	Pyruvic oxime dioxygenase homolog PODh	5.30	1.69E−08	1896.0	48.5
AFA2_02357	Nitrite reductase accessory protein NirV	− 2.28	0.02937	379.0	1843.2
AFA2_02614	Outer membrane protein	− 2.78	0.00454	282.6	1953.4
AFA2_02843	Flagellin	− 3.67	9.34E−05	639.6	8159.2
AFA2_03346	Aminobenzoate oxygenase/hydroxylamine oxidase DnfA	2.48	0.01329	3688.9	664.2
AFA2_03347	Ferredoxin DnfB	2.57	0.00909	2007.2	339.3
AFA2_03348	Glutamine amidotransferase DnfC	2.44	0.01566	2472.4	456.5
AFA2_03446	Phosphoadenosine phosphosulfate reductase	− 2.29	0.02805	240.3	1184.5
AFA2_03447	Sulfate adenylyltransferase subunit 2	− 2.69	0.00647	159.0	1033.8

LowC/Nexp cell compared to HighC/Nexp cells: 28 differentially expressed genes with FDR < 0.05 and logFC ≥ 2.0 or logFC ≤ -2.0 and either TPM > 1000 were extracted. The 28 genes are listed with their products with annotations, LogFC, FDR, and TPMs of LowC/Nexp and HighC/Nexp cells.

Results of differential expression analysis of the *A. faecalis* genes between the LowC/Nexp cells and the LowC/Nsta cells are shown in supplementary Table S3 and by MA plotting (supplementary Fig. S2b). A significant difference in the gene expression occurred between the LowC/Nsta cells and the LowC/Nexp cells; 182 genes were up-regulated and 349 genes were down-regulated in the LowC/Nsta cells compared to the LowC/Nexp cells. In the total 531 genes that showed differential expression between the LowC/Nexp cells and the LowC/Nsta cells, 66 and 43 genes were highly expressed (TPM > 1000) in the LowC/Nexp cells or the LowC/Nsta cells, respectively (supplementary Table S3). During the transition from the exponential growth phase to the stationary phase of the bacterial cells, expression of the *pod* and the *podh* gene clusters were significantly down-regulated in the LowC/Nsta cells compared to those in the LowC/Nexp cells. TPMs of the genes in the *suf* and the *dnf* clusters also tended to decrease in the LowC/Nsta cells compared to those of the LowC/Nexp cells. In addition, expression levels of denitrifying enzymes, NirK, NorB, and NosZ, the denitrification-related proteins such as cytochrome *c* (AFA2_02351), nitrite reductase accessory protein NirV (AFA2_02357), and N_2_O reductase regulator (AFA2_02247), were decreased in the LowC/Nsta cells. Expression of the genes involved in energy production by oxidative phosphorylation, such as a respiratory terminal enzyme, *cbb*_3_-type cytochrome *c* oxidase, and FoF1-ATP synthase, were inactivated significantly, and the genes of pyruvate dehydrogenase and NADH dehydrogenase also showed the same trend. Expression levels of 32 genes for ribosomal biogenesis and translation, and those of 47 genes for flagellar motility and chemotaxis, also decreased in the LowC/Nsta cells.

**Figure 4:**
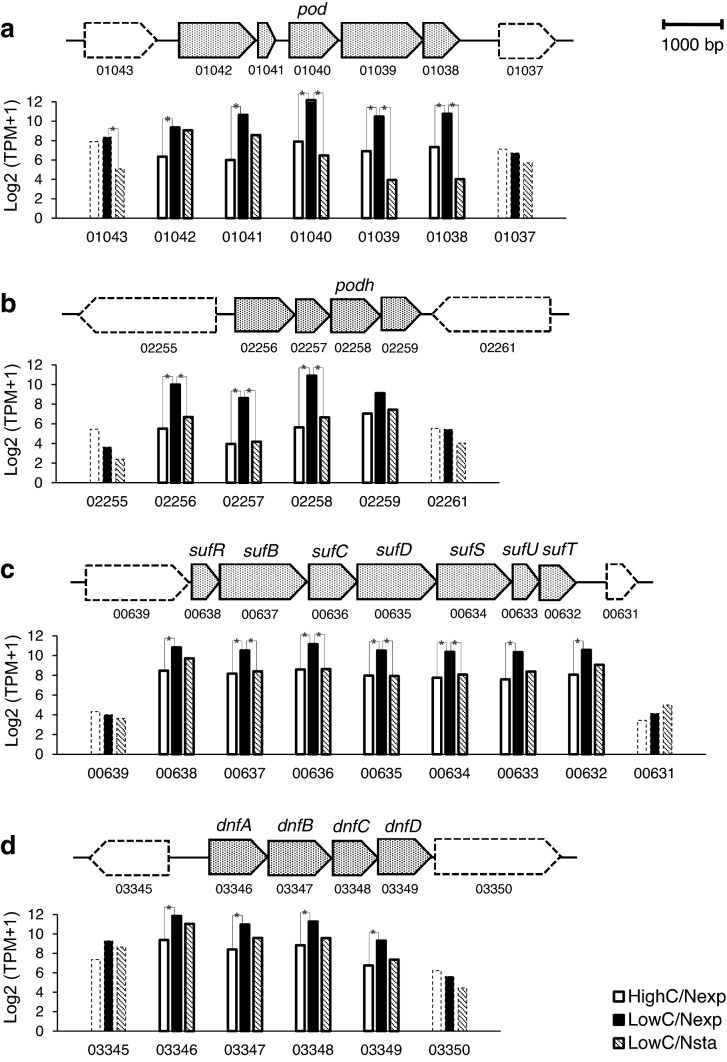
Arrangement of gene clusters whose expression levels significantly increased during induction of heterotrophic nitrification. The *pod* gene, *podh* gene, *suf* genes, and *dnf* genes constitute a polycistronic transcription unit (colored dots). Log_2_(TPM + 1) of each gene in HighC/Nexp, LowC/Nexp, and LowC/Nsta cells are indicated by white, black, and hatched bars, respectively. Asterisks designate the differential expression of the genes (logFC ≥ 2 or ≤ 2, and FDR < 0.05) between HighC/Nexp vs LowC/Nexp, and between LowC/Nsta vs LowC/Nexp. Numbers refer to the locus tags of the proteins; for example, 01040 indicates the gene with locus tag AFA2_01040.

As mentioned above, when *A. faecalis* was cultivated under Low C/N and High C/N conditions in which nitrifying activities are induced or not induced, respectively, expression levels of genes involved in the primary metabolic pathway for energy generation did not vary significantly, while a small number of genes including four gene clusters, *pod*, *podh*, *suf*, and *dnf*, were strongly activated in the exponential growth cells cultivated in the Low C/N condition. Gene arrangements of the four clusters and the TPM value of the genes in the LowC/Nexp, LowC/Nsta, and HighC/Nsta cells of *A. faecalis* are indicated in Fig. 4. As shown in Fig. 4a, all five genes (AFA2_01038-01042) including the *pod* gene (AFA2_01040) were up-regulated only in the LowC/Nexp cells, indicating that the gene cluster is transcribed as a single mRNA. Transcriptions of the *podh* gene cluster (AFA2_02256-02259), the *suf* gene cluster (AFA2_00632-00638), and the *dnf* gene cluster (AFA2_03346-03349) were also regulated similarly to that of the *pod* gene cluster, as shown in panels b–d in Fig. 4, respectively. These results suggest that transcriptions of the four gene clusters, which may play significant roles in the heterotrophic nitrification, are controlled by an identical regulatory system. Three genes encoding a putative transcription regulator were identified in the 105 genes showing differential expression between the LowC/Nexp cells and the HighC/Nexp cells. The SufR regulator (AFA2_00638), involved in the *suf* gene cluster in the *A. faecalis* genome, has been known to function as an iron-sulfur cluster-dependent repressor that regulates the iron-sulfur cluster biosynthesis by the SUF system in cyanobacteria^25^. Another two regulators, the TetR-family regulator (AFA2_02004) belonging to the large family of one-component signal transduction proteins, and the ArsR-family regulator (AFA2_03010) assigned as an arsenate-dependent repressor, were down-regulated in the LowC/Nexp cells. The regulatory system that controls transcription of the four gene clusters and thereby induction of heterotrophic nitrification remains unknown at present. The possibility that these transcription factors are involved in the regulation of heterotrophic nitrification should be investigated.

In the heterotrophic nitrifying bacteria as well as in autotrophic ammonia-oxidizers, the first step of nitrification has been regarded as the oxidation of ammonia to hydroxylamine catalyzed by AMO^20^. Moir et al.^26^ isolated a cosmid clone of *Paracoccus denitrificans* that shows high nitrifying activity by homologous expression screening, and succeeded in the purification of AMO composed of two subunits whose molecular masses are 38 and 46 kDa from the strain. It has been shown that, like the *Nitrosomonas europaea* AMO, the *P. denitrificans* enzyme is sensitive to visible light and has the activity to oxidize ethylene to epoxyethane^27^. Unfortunately, there is not yet evidence that the purified AMO from *P. denitrificans* is a product of the putative *amo* gene (Pden_0324 or Pden_4953). More recently, purification of AMO with a molecular mass of 31 kDa from *Acinetobacter* sp. Y16 was reported, but its nucleotide sequence has not been published^28^. The putative *amoA* gene (AFA2_02284) is present the *A. faecalis* genome. Phylogenetic analysis indicates that the putative AmoA of *A. faecalis* forms a cluster with those of other heterotrophic nitrifiers, and is deeply branched from those of autotrophic ammonia oxidizers in the betaproteobacteria, gammaproteobacteria, comammox *Nitrospira*, and thaumarchaea (Supplementary Fig. S3). The up-regulation of the *amoA* gene in the LowC/Nexp cells (TPM = 7.7) that had been initially predicted was not observed, and its expression level was very low, similar to those in the HighC/Nexp (TPM = 10.0) and the LowC/Nsta cells (TPM = 4.6) as indicated in supplementary Tables S2 and S3. These results suggest that, even if the AFA2_02284 gene product possesses AMO activity, it is unlikely that it contributes significantly to the nitrifying activity in the LowC/Nexp cells of *A. faecalis*. The *dnfABCD* genes, which were strongly up-regulated in the LowC/Nexp cells, are involved in the putative DNF pathway, a novel biochemical process that converts hydroxylamine to N_2_ gas proposed recently by Wu et al.^24^. The authors also indicated that the DnfA alone could catalyze the DNF reaction in the presence of O_2_ and an appropriate artificial reducing reagent, and the activity was not affected by supplementation of DnfB and/or DnfC recombinants into the reaction solution. DnfC is annotated as a glutamine amidotransferase which catalyzes the transfer of the amide group from glutamine to a specific substrate. Here it should be noted that glutamine is a primary product of ammonia assimilation process, in which ammonia is incorporated into the amide group of glutamine catalyzed by glutamine synthetase (AFA2_00555, TPM = 3457 in the LowC/Nexp cells). A scenario in which the hydroxylamine is generated from the amide group of glutamine by an unknown biochemical process involving the DNF enzymes rather than the AMO reaction is attractive and should be explored in the future.

Hydroxylamine reacts non-enzymatically with pyruvate to form pyruvate oxime. POD catalyzes dioxygenation of pyruvic oxime and forms nitrite and pyruvate as the final step of heterotrophic nitrification^20,21^. For the enzymatic characterization of the *podh* gene product, POD activity was measured using a recombinant PODh expressed in *Escherichia coli*. The PODh showed the POD activity, while its apparent rate constant (0.26 s^-1^) was only 5.5% of that of POD, suggesting a limited contribution to the nitrite production in the heterotrophic nitrification in *A. faecalis* (supplementary Table S4).

The SUF system is an iron-sulfur cluster biogenetic pathway that is widespread in bacteria, archaea, and chloroplasts^29^. In *E. coli*, it has been known that an iron-sulfur cluster biogenesis by the SUF system is activated in response to stresses such as iron starvation, oxidative stress, and heavy metal stress, and plays a complementary role to the housekeeping ISC (iron sulfur cluster) biosynthetic pathway^30^. The *iscRSUA* gene cluster (AFA2_00926-00923), encoding the components of the ISC machinery, is also identified in the *A. faecalis* genome. Expression levels of the *isc* genes did not change significantly between the LowC/Nexp cells and the HighC/Nexp cells (supplementary Table S2). In the LowC/Nexp cells, expression of various iron-sulfur proteins, including DnfB, the putative electron carrier from NADH to DnfA, might be promoted by the activated SUF system. It remains unclear how the SUF system is overtly involved in the heterotrophic nitrification process. The possibility that cultivation in the nitrogen-rich medium is stressful enough for activation of the SUF system, and that the SUF system may not be directly involved in the biochemical machinery of heterotrophic nitrification should also be considered.

Not only nitrite, but also nitrate was accumulated in the low C/N medium, suggesting the presence of a reaction pathway that oxidizes nitrite to nitrate in *A. faecalis* (Fig. 3). On the *A. faecalis* genome, there are no genes that are homologous to the nitrite oxidase gene found in the autotrophic nitrite-oxidizing bacteria. Catalase-like nitrite oxidase (NiOx) has been reported in the heterotrophic nitrifying microorganisms *Aspergillus flavus, Candida rugosa,* and *Bacillus badius*^31–33^. The gene (AFA2_01914) encoding a catalase-like protein which shows sequence homology to the NiOx in *B. badius* 1–73 was found on the *A. faecalis* genome. The nitrate accumulated in the medium may have been produced by the enzymatic activity of this gene product.

Based on the genetic structure, it had been expected that the *pod* gene together with its neighboring genes would constitute a polycistronic transcription unit. Transcriptome analysis demonstrated that the genes encoding 2-hydroxychromene-2-carboxylate (HCCA) isomerase (AFA2_01038), metabolite/proton symporter (MHS)-family transporter (AFA2_01039), a hypothetical protein (AFA2_01041), and N-acetyltransferase (AFA2_01042) were simultaneously up-regulated with the *pod* gene in the LowC/Nexp cells as predicted (Fig. 4a). Similarly, simultaneous activation of the genes encoding the PODh, a TctC homolog of the tripartite tricarboxylate transporters (TTT)-family (AFA2_02256), and a glutathione S-transferase (GST) with unknown function (AFA2_02257) also observed in the LowC/Nexp cells (Fig. 4b). HCCA isomerase, a member of the kappa class GST, in the *pod* gene cluster is an enzyme participating in a degradation pathway for naphthalene^34^. A GST gene is also present in the *podh* gene cluster. Bacterial GST is known to associate with various metabolic processes, especially the degradation and detoxification of persistent organic compounds such as naphthalene^35^. The MHS-family transporter is a subfamily of the major facilitator superfamily transporter, and some of them have been suggested to be involved in the transport of aromatic compounds^36,37^. TctC homologs of the TTT-family are present in a variety of bacterial species, and many of them are found near the gene clusters associated with specific biochemical functions, such as degradation of aromatic compounds^38^.

It has been known that the *pod* genes are distributed widely in the microorganisms of the phyla Proteobacteria and Actinobacteria, and the eukaryotic phylum Ascomycota^21^. Many *pod* genes present in the proteobacteria are forming a gene cluster with the GST gene and the transporter-related gene as are the *pod* and *podh* genes in *A. faecalis*. We cannot deny a possibility that the *pod* clusters with this gene configuration may associate not only with the POD activity but with the uptake and degradation of external substrates such as aromatic compounds. In this situation, the enzymatic function of the POD and the physiological implication of heterotrophic nitrification should be re-examined in a future study.

## Materials and methods

### Cultivation conditions of Alcaligenes faecalis

*A. faecalis* NBRC13111 was cultivated in a synthetic medium containing ammonium chloride and organic acid (sodium pyruvate or sodium succinate) as sole nitrogen and carbon sources, respectively, according to Ono et al.^19^ with slight modifications. The synthetic medium composed of 10.8 g/L K_2_HPO_4_, 0.53 g/L KH_2_PO_4_, 0.20 g/L MgSO_4_·7H_2_0, 38.9 mg/L CaCl_2_, 10.0 mg/L FeSO_4_·7H_2_O, 1.0 mg/L Na_2_MoO_4_·2H_2_O, 2.0 mg/L MnCl_2_·4H_2_O, 0.02 mg/L CoCl_2_·6H_2_O, 1.0 mg/L ZnSO_4_·7H_2_O, and 1.0 mg/L CuSO_4_·5H_2_O was prepared, then the pH of the medium was confirmed to be 7.8. After autoclaving, the stock solutions of ammonium chloride and organic acids were added to the medium to reach appropriate concentrations using a syringe-driven filter unit of 0.22 µm pore size (Merck KGaA, Darmstadt, Germany). Precultivation of *A. faecalis* was carried out in 3 mL of 2× YT medium at 37 °C with shaking at 180 rpm for 21 h. Bacterial cells were collected from 0.5 mL of the medium by centrifugation at 5000×*g* for 10 min using a refrigerated centrifuge (model 3700, Kubota Co. Ltd., Tokyo, Japan), and the pellet obtained was suspended in the synthetic medium, then centrifuged again under the same conditions. The resulting cell pellet was inoculated into 100 mL of the synthetic medium containing ammonium and an organic acid, then the cultivation experiment was started with shaking at 120 rpm at 30 °C. OD_600_ was measured in a 1 cm light-path cuvette using a UV-2600 spectrophotometer (Shimadzu, Kyoto, Japan).

### Measurement of heterotrophic nitrification activity

A part of the cultivation medium was sampled and centrifuged at 5000×*g* for 10 min, then the supernatant obtained was used to determine the concentrations of ammonia, nitrite, and nitrate. The ammonia concentration was assayed spectrophotometrically by the indophenol blue method using the Ammonia Test Wako (Fujifilm Wako Pure Chemical Co., Osaka, Japan). Concentrations of nitrite and nitrate were measured spectrophotometrically by a diazo-coupling method^39^ and a brucine method^40^, respectively. POD activity was determined by measuring the rate of nitrite production in the assay solution containing 20 mM Tris–HCl buffer (pH 8.0), 1.0 mM sodium ascorbate, and 1.0 mM pyruvic oxime. Pyruvic oxime was synthesized according to Quastel et al*.*^41^. The protein concentration was measured using a BCA protein assay kit (Pierce, Rockford, IL) with bovine serum albumin as the standard, and the specific activity was calculated.

### Preparation of bacterial cells and extraction of total RNA

*A. faecalis* was cultivated aerobically in the synthetic medium containing 5 mM ammonium chloride and 10 mM sodium pyruvate (LowC/N medium), of which the C/N ratio was 5. After starting cultivation in the LowC/N medium, bacterial cells in the mid-exponential growth phase, designated “LowC/Nexp” cells, and those in the stationary phase, designated “LowC/Nsta” cells, were harvested at 18 h and 28 h, respectively. Cells were also cultivated using the synthetic medium containing 5 mM ammonium chloride and 40 mM sodium pyruvate (HighC/N medium), of which the C/N ratio was 20. Bacterial cells in the mid-exponential growth phase, designated “HighC/Nexp” cells, were harvested at 18 h after starting cultivation in the HighC/N medium. The cells were pelleted by centrifugation at 5000×*g* for 10 min using a refrigerated centrifuge, then an RNA*later* (Ambion, Carlsbad, CA) was layered on the pellet and was stored at − 80 °C until experimental use.

After thawing the three samples, the supernatant was removed by centrifugation at 5000×*g* for10 min, then the total RNA was extracted from each sample of bacterial cells using the PureLink™ RNA Mini Kit (Thermo Fisher Scientific, Waltham, MA), according to a recommended protocol. The genomic DNA contaminated in the extracted total RNA solution was removed by DNase treatment using the Turbo DNA-*free* Kit (Thermo Fisher Scientific). The concentration of the nucleic acid was measured spectrophotometrically using BioSpec-nano (Shimadzu) and Qubit Fluorometer (Thermo Fisher Scientific).

### RNA sequencing and differential gene expression analysis

Ribosomal RNA molecules were removed from the total RNA of the three samples using a Ribo-Zero rRNA Removal Kit for bacteria (Illumina, Inc., San Diego, CA), and the rRNA-depleted RNA was purified using the RNeasy MinElute Cleanup Kit (Kapa Biosystems, Inc., Woburn, MA). RNA-seq libraries, LowC/Nexp, LowC/Nsta, and HighC/Nexp were constructed using a KAPA Stranded mRNA-seq Kit (Kapa Biosystems) according to the manufacturer’s instructions with 14 cycles of library amplification except for skipping an mRNA capture step. The three libraries were sequenced using a HiSeq 4000 platform (Illumina) to generate 2 × 101-bp paired-end sequence reads at Macrogen, Inc. (Seoul, South Korea).

The following sequence data processing was done separately on the three libraries. The raw reads were cleaned up using Trimmomatic ver. 0.36 by removing adapter sequences and low-quality reads with the following parameters: CROP, 100; SLIDINGWINDOW, 4; QUALITY, 15; MINLEN, 75^42^. The adapter-trimming and quality-filtered reads were aligned to the draft genome sequence of *A. faecalis subsp. faecalis* NBRC 13111 (GenBank accession number BDHG01000000) using HISAT2 ver. 2.2.0 with a no-spliced-alignment option^43^. Read counts were calculated from the BAM files using featureCounts ver. 2.0.0^44^, and TPM values were calculated to normalize gene lengths and total read counts.

Differential expression analysis of the LowC/Nexp and HighC/Nexp, and the LowC/Nexp and the LowC/Nsta was performed using the edgeR package ver. 3.16.5^45^, which is capable of the detection of differentially expressed genes (DEGs) with no replicates, as previously described^46^. DEGs were defined by the logFC ≥ 2 or ≤ 2, and FDR < 0.05.

### Other experiments

All protein sequences in *A. faecalis* NBRC13111 were annotated using KofamKOALA to predict functions^47^. Homology search and phylogenetic analysis were performed using Blast (http://blast.genome.jp/) and MEGA X^48^, respectively. All chemicals used in the experiments were of the highest grade commercially available.

## Supplementary Information

Supplementary Figure S1.

Supplementary Figure S2.

Supplementary Figure S3.

Supplementary Table S1.

Supplementary Table S2.

Supplementary Table S3.

Supplementary Table S4.

Supplementary Information 8.

## Data Availability

Raw reads for RNA-seq analyzed in this study have been deposited in the DDBJ Sequence Read Archive (DRA) under the accession numbers DRR284713 (LowC/Nexp), DRR284714 (LowC/Nsta), and DRR284715 (HighC/Nexp).

## Abbreviations

AOA: Ammonia-oxidizing archaea
AOB: Ammonia-oxidizing bacteria
AMO: Ammonia monooxygenase
HAO: Hydroxylamine oxidoreductase
POD: Pyruvic oxime dioxygenase
C/N: Carbon/nitrogen
OD_600_: Optical density at 600 nm
*t*_D_: Doubling time
TPM: Transcripts per million
logFC: Log2 fold change
FDR: False discovery rate
DNF: Dinitrogen formation
SUF: Sulfur formation
ISC: Iron sulfur cluster
NiOx: Catalase-like nitrite oxidase
GST: Glutathione S-transferase
S.E.: Standard error
