# Supplementary material for: Gene expression analysis of Alcaligenes faecalis during induction of heterotrophic nitrification
Source: Sci Rep. 2021 Nov 29;11:23105. doi: 10.1038/s41598-021-02579-3 (PMC8629993; doi:10.1038/s41598-021-02579-3)
Supplement: Supplementary file 1 — Supplementary Figure S1. [file 41598_2021_2579_MOESM1_ESM.docx]

**Supplementary Fig. S1.** **Cultivation of *A. faecalis* for transcriptome analysis.** *A. faecalis* was cultivated in a synthetic medium containing 5 mM ammonium chloride as the only nitrogen source, and 10 mM **(a)** or 40 mM **(b)** sodium pyruvate as the only carbon source. The culture medium was sampled, and the time-course of growth (OD_600_, open circles) was measured. The concentrations of nitrite (hatched bar) were measured after removing bacteria cells from the culture medium by centrifugation. Total RNA was extracted from bacterial cells at the exponential (18 h) and stationary (28 h) growth phases of (**a**) and at the exponential (18 h) growth phase of (**b**) for transcriptome analysis (LowC/Nexp and LowC/Nsta, HighC/Nexp, respectively).
