# Supplementary material for: Gene expression analysis of Alcaligenes faecalis during induction of heterotrophic nitrification
Source: Sci Rep. 2021 Nov 29;11:23105. doi: 10.1038/s41598-021-02579-3 (PMC8629993; doi:10.1038/s41598-021-02579-3)
Supplement: Supplementary file 2 — Supplementary Figure S2. [file 41598_2021_2579_MOESM2_ESM.docx]

**Supplementary Fig. S2. MA plot of differentially expressed genes.** Comparisons of LowC/Nexp cells to HighC/Nexp cells **(a)** and LowC/Nsta cells to LowC/Nexp cells **(b)** by differential analysis. The genes in the *pod* cluster (AFA2_01038–01042) and in the *podh* cluster (AFA2_02256–02259) are indicated by blue and red, respectively, in each panel. The genes in the *suf* cluster (AFA2_00632–00638) and the *dnf* cluster (AFA2_03346–03349) are indicated by purple and green, respectively. Denitrifying genes, *nirK* (AFA2_02352), *norB* (AFA2_02353), and *nosZ* (AFA2_02246) are indicated by yellow. Differentially expressed genes (FDR <0.05 and logFC ≥2.0 or ≤-2.0) excluding these genes are plotted in orange.
