# Supplementary material for: Gene expression analysis of Alcaligenes faecalis during induction of heterotrophic nitrification
Source: Sci Rep. 2021 Nov 29;11:23105. doi: 10.1038/s41598-021-02579-3 (PMC8629993; doi:10.1038/s41598-021-02579-3)
Supplement: Supplementary file 3 — Supplementary Figure S3. [file 41598_2021_2579_MOESM3_ESM.docx]

**Supplementary Fig. S3. Phylogenetic relationship of putative AmoA from heterotrophic nitrifier with other AmoA and PmoA.** Neighbor-joining tree drawn by the MEGA X program was revealed using the amino acid sequences of AmoA from heterotrophic nitrifier, ammonia-oxidizing bacteria (AOB), ammonia-oxidizing archaea (AOA) and complete ammonia oxidizer (comammox), and particulate methane monooxygenase subunit A (PmoA) from methane oxidizer. Accession numbers of the amino acid sequence of AmoA and PmoA are shown in parentheses. The percentage of replicate trees in which the associated taxa clustered together in the bootstrap test (500 replicates) are shown next to the branches. The scale bar represents the number of amino acid substitutions per site.
