# Supplementary material for: Gene expression analysis of Alcaligenes faecalis during induction of heterotrophic nitrification
Source: Sci Rep. 2021 Nov 29;11:23105. doi: 10.1038/s41598-021-02579-3 (PMC8629993; doi:10.1038/s41598-021-02579-3)
Supplement: Supplementary file 8 — Supplementary Information 8. [file 41598_2021_2579_MOESM8_ESM.docx]

**SUPPLEMENTARY MATERIALS**

**Construction of a PODh overexpression vector**

Oligonucleotide primers for PCR amplification of the gene encoding *A. faecalis* PODh, AfpodhF (5’-TCA TAT GAG TGA CAC CAT GAG CTT GTC-3’, artificial *Nde*I recognition site underlined), and AfpodhR (5’-TCT CGA GCT CAA AAA CAT CCG TAT GGC-3’, artificial *Xho*I recognition site underlined), were designed according to its nucleotide sequence. Standard protocols used for handling *E. coli* DNA followed Sambrook and Russell (2001). Amplification was carried out using KOD-plus DNA polymerase (Toyobo, Osaka, Japan) and *A. faecalis* genomic DNA as a template. The 786 bp PCR product obtained was cloned into a pCR-blunt TOPO II vector (Invitrogen, Carlsbad, CA), yielding pCRAfPODh. Site-directed mutagenesis for codon optimization was carried out by technical application of PCR. To remove the pre-existing internal *Nde*I-recognition site in the *podh* gene, PCR amplification was carried out again using the pCRAfPODh plasmid as a template and a set of oligonucleotides, Afpodh^T420C^F (5'-ATG TAT CCC ACA TGG ACA ACT-3', mutation position underlined) and Afpodh^T420C^R (5'-AGT TGT CCA TGT GGG ATA CAT-3') as primers for replacing the corresponding CAT (His140) with CAC. After treatment with restriction enzyme *Dpn*I (Takara Shuzo Co. Ltd., Kyoto, Japan) to decompose the template DNA, the PCR product was introduced into the *E. coli* JM109 cells. The PCR product was cyclized by homologous recombination between the 5’ and 3’ regions in the host cells, yielding pCRAfPODh420C. After confirmation of the nucleotide sequence, the *podh*^T420C^ gene was cloned into a pCR-blunt TOPO II vector, yielding pCRAfPODh^T420C^. The insert of pCRAfPODh^T420C^ was digested with both *Nde*I and *Xho*I, and then cloned into the same restriction site of a pET21a^+^ expression vector (Novagen, Darmstadt, Germany), yielding the expression plasmid pAfPODh^T420C^. The pAfPODh^T420C^ plasmid was introduced into *E. coli* BL21(DE3)-CodonPlus (Agilent Technologies, Santa Clara, CA), generating strain *Af*PODh for overexpression of the recombinant PODh.

**Purification and enzymatic analysis of recombinant PODh**

*E. coli* strain *Af*PODh was cultivated aerobically in 2×YT medium (1 L) supplemented with 50 μg/mL ampicillin at 37°C with reciprocal shaking at 150 rpm. In a mid-exponential growth stage (OD_600_ = 0.6−0.8), 0.1 M stock solution of isopropyl β-D-1-thiogalactopyranoside (IPTG) was added to the medium to reach 0.4 mM for induction of the PODh. After incubation at 20°C with shaking at 150 rpm for 3 h, the cells were collected by centrifugation. Cultivated AfPh cells were suspended in 25 mM sodium phosphate (pH 7.4, buffer A). The suspension was sonicated using a VP-30S supersonic oscillator (Taitec Co., Ltd, Saitama, Japan) for 30 × 10 sec at full power to disrupt cells. After removing unbroken cells by centrifugation at 12,000 × *g* for 10 min, the supernatant obtained was centrifuged at 140,000 × *g* for 65 min using an Optima L-90K ultracentrifuge (Beckman Coulter, Inc., Brea, CA). The soluble fraction thus obtained was applied to a Ni^2+^ affinity column (1 × 2 cm) of Chelating Sepharose^TM^ Fast Flow (GE Healthcare, Buckinghamshire, UK) that had been equilibrated with buffer A. The recombinant protein adsorbed on the column was eluted by buffer A containing 500 mM imidazole after sequential washing by buffer A containing 0.5 M sodium glutamate, 20 mM imidazole, and 50 mM imidazole. The fractions that showed POD activity were collected, then dialyzed three times against buffer A containing 250 mM NaCl for 1 h at 4°C. The resulting solution was used as the purified sample for experiments after confirmation of an electrophoretically homogeneous state by SDS-PAGE (Schägger & von Jagow 1987). POD activity of the purified recombinant protein was measured, and the apparent rate constant (*k*_cat_) and affinity constant (K_m_ for pyruvic oxime) were determined according to the previous report (Tsujino *et al*. 2017).

**References**

Sambrook, J. & Russell, D. W. *Molecular Cloning: A Laboratory Manual*, 3rd ed. (Cold Spring Harbor Laboratory Press, 2001).

Tsujino, S., Uematsu, C., Dohra, H. & Fujiwara, T. Pyruvic oxime dioxygenase from heterotrophic nitrifier *Alcaligenes faecalis* is a nonheme Fe^(II)^-dependent enzyme homologous to class II aldolase. *Sci. Rep.* **7**, 39991; 10.1038/srep39991 (2017).

Schägger, H. & von Jagow, G. Tricine-sodium dodecyl sulfate-polyacrylamide gel electrophoresis for the separation of proteins in the range from 1 to 100 kDa. *Anal. Biochem.* **166**, 368–379 (1987).
